# Ion toxicity in waterlogged soils: mechanisms of root response and adaptive strategies

**DOI:** 10.3389/fpls.2025.1653008

**Published:** 2025-08-15

**Authors:** Lin Zhang, Yan Li, Yanqin Wang, Zhaohui Liu, Herbert J. Kronzucker, Zhaoyue Wang, Weiming Shi, Guangjie Li

**Affiliations:** ^1^ State Key Laboratory of Nutrient Use and Management, National Agricultural Experimental Station for Soil Quality, Jinan, China, Key Laboratory of Agro-Environment of Huang-Huai-Hai Plain, Ministry of Agriculture and Rural Affairs, Institute of Agricultural Resources and Environment, Shandong Academy of Agricultural Sciences, Jinan, China; ^2^ School of Biological Sciences, University of Western Australia, Perth, WA, Australia; ^3^ State Key Laboratory of Soil and Sustainable Agriculture, Chinese Academy of Sciences Institute of Soil Science, Nanjing, China

**Keywords:** waterlogging, ion toxicity, root architecture, signaling, Arabidopsis

## Abstract

Waterlogging poses a significant global threat to agriculture by inducing ion toxicities (e.g. Fe²^+^, Mn²^+^, NH_4_^+^) in roots due to soil redox changes. This review synthesizes current insights into how plant roots, particularly in Arabidopsis, respond to these toxicities, focusing on root system architecture (RSA) modifications and underlying mechanisms. Under waterlogging, soil redox changes drive Fe²^+^ and Mn²^+^ accumulation in reducing layers, while NH_4_^+^-based fertilizers elevate NH_4_^+^:NO_3_^-^ ratios. NH_4_^+^ inhibits primary root (PR) elongation by disrupting cell division and energy metabolism via VTC1 and LPR2 genes, while locally stimulating lateral root (LR) formation through pH-dependent auxin diffusion. Ethylene and NO signaling interact to modulate gravitropism via PIN2 and ARG1/GSA1 pathways. Fe toxicity arrests PR growth by reducing cell activity in the root tip, involving ethylene, ROS (H_2_O_2_/O_2_^-^), and NO pathways. GSNOR emerges as a key gene for Fe tolerance, balancing NO homeostasis. LR formation under Fe stress relies on PIN2/AUX1-mediated auxin transport and ferritin storage, with ROS-auxin crosstalk influencing adaptive responses. Mn toxicity inhibits PR elongation by repressing auxin biosynthesis (YUC genes) and efflux (PIN4/PIN7), while miR781 and cation transporters (CAX4, MTP11) facilitate detoxification. Vacuolar compartmentation and Ca²^+^ signaling via ECA proteins are also critical. Despite progress, key gaps remain: identifying ion sensors in root tips, extrapolating findings to long-lived species, modeling multi-ion interactions under dynamic waterlogging conditions, and establishing real-time root signal monitoring systems. Integrating temporal and environmental factors (e.g. temperature) will enhance understanding of RSA reprogramming for waterlogging tolerance.

## Introduction

Waterlogging is a serious worldwide environmental problem, which restricts agricultural production especially in low-lying rain-fed areas. The problem is expected to worsen due to climate change in the coming decades, and this trend is already evidenced in the increased frequency of devastating floods in more recent history (Seneviratne et al., 2012; Zhang et al., 2021b). According to the United Nations’ Food and Agriculture Organization (FAO), floods were responsible for nearly two-thirds of global crop losses between 2006 and 2016, resulting in losses valued in the billions of US dollars (FAO, 2018). In the United States, floods are also a serious hazard and, over the 12-year period from 2000 to 2011, have been ranked as the second leading abiotic stressor causing crop yield losses, second only to drought (Bailey-Serres et al., 2012). Waterlogging pressures have also increased in frequency in countries such as Australia and China, due to factors such as heavy rains, poor soil structure, inadequate drainage systems, and compacted subsoil (Samad et al., 2001; Collaku and Harrison, 2002; Tong et al., 2021). NASA (National Aeronautics and Space Administration) simulation models have predicted crop production losses approximating $3 billion USD per year by 2030, based on current extreme-weather trends associated with global climate change (Rosenzweig et al., 2002; Kaur et al., 2020).

Waterlogged soils are typically low in oxygen (O_2_), while plant roots require oxygen for respiration, to maintain nutrient acquisition, and also to prevent ion toxicities (Kronzucker et al., 1998). Adverse effects associated with anoxic or hypoxic soil conditions on roots will affect shoot growth and crop yield (Fukao et al., 2019). There have been extensive proposals on the mechanisms of waterlogging or flooding tolerance and hypoxia stress (Zhang et al., 2025). Setter and Waters (2003) discuss twenty-two tolerance mechanisms, including mechanisms pertaining to phenology, morphology, nutrition, and root metabolism. Additionally, plants may suffer from ion toxicities due to a decline in soil redox potential under prolonged flooding conditions, leading to elevated concentrations of certain potentially toxic ions (e.g. Mn^2+^, Fe^2+^) in the waterlogged soil solution (Setter et al., 2009). A further decline in available O_2_ concentration impairs soil microbial activity, ultimately reducing the abundance of oxidized species (e.g., NO_3_^−^, SO_4_^2−^, Fe^3+^) while elevating levels of reduced compounds (e.g., Mn^2+^, Fe^2+^, H_2_S, NH_4_^+^). Additionally, the reduction of Fe and Mn oxides can facilitate the release of other non-redox-sensitive elements, including Co, P, Ni, Cd, and Zn (Li et al., 2012). When the redox potential drops below -150 mV, SO_4_^2−^ is reduced to H_2_S (Hydrogen sulfide), a compound also toxic to plants (Frohne et al., 2011). In most plants, prolonged exposure to elevated H_2_S levels induces PR inhibition and leaf injury, followed by leaf abscission, and ultimately leads to overall impairment of plant growth (Arif et al., 2021; Li et al., 2022b). Waterlogging stress, due to hypoxia, causes significant changes in soil physical and chemical properties (Harvey et al., 2019), reducing not only the soil oxidation–reduction state (Eh) but also altering its elemental profile (Rinklebe and Shaheen, 2017). The availability of nutrients depends on the physical, chemical, and biological properties of the soil, especially Eh. The sequence of electron-acceptor reduction is as follows: oxygen reduction begins at < 550 mV, and Mn^4+^ reduction follows at < 500 mV. Below 330 mV, soil is characterized by the complete absence of oxygen. Any further decline of the Eh below < 200 mV results in reduction of Fe^3+^ to Fe^2+^. At lower pH (pH ≤ 5), a redox potential of only +309 mV is sufficient to facilitate a build-up of toxic Fe^2+^ levels to ~300 ppm (Onaga et al., 2016). Indeed, microelement toxicities, predicted from soil analyses during waterlogging, have been substantiated by plant analyses for wheat and rice grown in waterlogged soils in the USA, Australia, China, and India. For plants growing in waterlogged acidic soil, the content of Mn and Fe in the plant increases 2- to 10- times compared with that in drained soil, exceeding critical toxicity thresholds for many species (Khabaz-Saberi et al., 2010a, b, 2012).

When the soil becomes waterlogged, roots need to continue functioning so as to sustain the shoots with nutrients and water. The root system is the centre of “communication” between the plant and the rhizosphere. The root system is also the direct contact site for ions in soil, and ion toxicities caused by waterlogging often limit the formation of aerenchyma, aggravating root hypoxia and inhibiting root growth (Pedersen et al., 2021). In studies on crop species growing in acidic soils in Australia, roots of wheat genotypes tolerant to elevated soil concentrations of Mn and Fe have been found to display superior growth than those of intolerant genotypes under waterlogging conditions (Khabaz-Saberi et al., 2010a, b, 2012). Enhanced vegetative growth and resistance to ion toxicities conferred improved recovery times after cessation of waterlogging and reduced potential yield losses (Jiménez et al., 2019). Root morphology and anatomical characteristics help plants adapt to waterlogging-induced ion toxicities. Plants can respond to ion toxicity conditions by changing the root growth architecture such as by altering root number and length, a process known as the stressed-induced morphogenic response (Shabala, 2011; Herzog et al., 2016; Daniel and Hartman, 2024).

Several recent review articles have addressed root adaptation to low-oxygen conditions and associated energy crises in Arabidopsis thaliana and other plant species (Manghwar et al., 2024; Zhang et al., 2025). However, limited coverage has been devoted to root growth inhibition caused by ion toxicities induced by waterlogging. Horst (1988) reported that wheat is among the most waterlogging-intolerant crops, with sensitive plants often suffering from iron (Fe) or manganese (Mn) toxicity under waterlogged conditions. Recent studies across laboratories have proposed that waterlogging tolerance typically arises from combined tolerance to anaerobiosis and elemental toxicities (e.g., Mn, Fe, and NH_4_^+^) in diverse soils.

In recent years, extensive research on plant roots, especially so in Arabidopsis, has investigated ion toxicities under reducing conditions to identify molecular determinants of tolerance to waterlogging-induced ion stresses. Here, we review recent advances in our understanding of how Arabidopsis roots respond to waterlogging-induced ion toxicities, with a particular focus on the underlying mechanisms. We highlight three representative and well-studied ion toxicities - Fe, Mn, and NH_4_^+^ - to illustrate how these stresses impact root morphology, including PR elongation, lateral root (LR) formation, and the adaptive significance of root-system-architecture (RSA) modifications in mitigating waterlogging-induced ion stresses.

## Responses of roots to NH4+ toxicity

**Figure 1:**
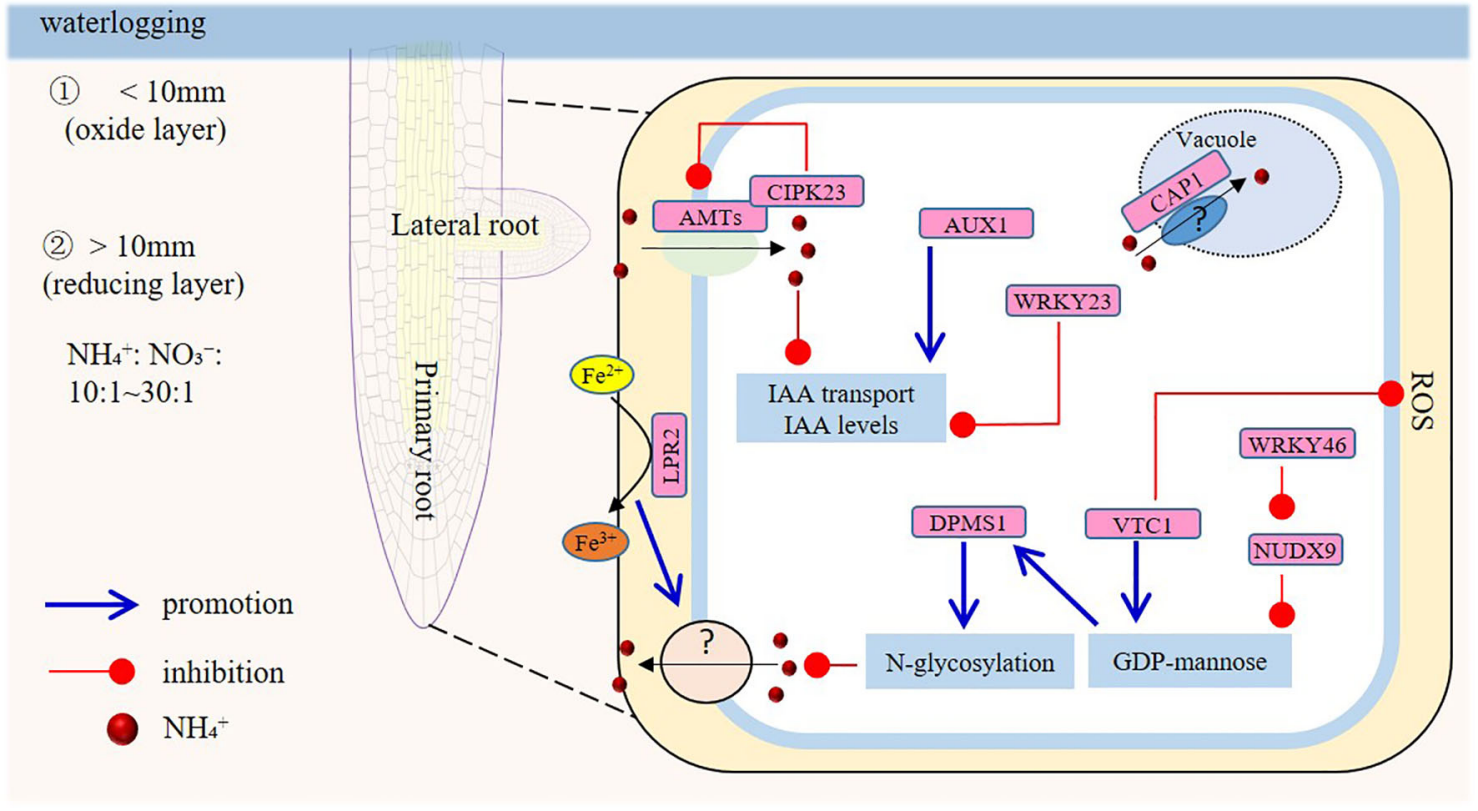
Model of the signaling responses in roots under ammonium (NH_4_^+^) toxicity. Long-term waterlogging partitions soil into two layers: a thin surface oxide layer (usually ≤10 mm thick) and a lower reducing layer. Upon application of ammonium-based fertilizers, the NH_4_^+^/NO_3_^-^ ratio in the reducing layer’s soil solution can hit 10:1. In chronically flooded soils, this ratio may surpass 30:1. Excess NH_4_^+^ restrains primary root growth by inhibiting meristem-cell proliferation at the root apex and by reducing cell size. The VTC1 and LPR2 genes are crucial; VTC1-mediated GDP-mannose synthesis affects protein glycosylation, and LPR2-regulated Fe accumulation impairs root growth. NUDX9 and DPMS1 are also involved in the GDP-mannose pathway. AMTs, CIPK23, AUX1, and WRKY23 play roles, though the function of auxin is controversial. Long-term NH_4_^+^ exposure suppresses lateral root formation systemically via shoot-derived signals involving ethylene and AUX1. Additionally, the CAP1 gene, related to vacuolar ammonium compartmentation, affects root hair sensitivity to high NH_4_^+^.

Long-term waterlogging divides soil into two distinct layers with contrasting properties: a thin surface oxide layer (typically ≤10 mm thick, often just a few millimeters) and a lower reducing layer, although oxygen presence in the direct vicinity of roots, through oxygen extrusion from living root tissue, can partially disrupt this pattern (Kirk and Kronzucker, 2005). When ammonium-based fertilizers (e.g., ammonium sulfate, ammonium bicarbonate, urea) are applied, the ratio of NH_4_^+^ to NO_3_^-^ in the reducing layer’s soil solution can reach 10:1, and in chronically flooded soils, this ratio may even exceed 30:1 (Nguyen et al., 2018; Parit et al., 2020). Recent research indicates that high NH_4_^+^ primarily impacts root system development, including PR elongation and LR branching, and that these effects are localized to distinct root tip regions (**Figure 1**). Root elongation inhibition appears to target the elongation zone. Conversely, short-term local NH_4_^+^ supply enhances LR density, a response potentially linked to NH_4_^+^ transporters.

### Primary root growth

Inhibition of PR and LR elongation is a common symptom of NH_4_^+^ toxicity, especially when NH_4_^+^ is the predominant nitrogen source (Britto and Kronzucker, 2002). The root-tip zone is also the key site for sensing NH_4_^+^, leading to inhibition of PR growth and LR elongation (Li et al., 2014; Coleto et al., 2023). NH_4_^+^ inhibits PR growth mainly by inhibiting cell proliferation of the root apical meristem and by reducing the cell size from the longitudinal part, while the structure and the activity of the stem cell niche are not affected (Li et al., 2010; Di et al., 2021). Several hypotheses have been proposed to explain the mechanisms of inhibition of root elongation by NH_4_^+^ in Arabidopsis, including increased energy consumption in roots, coupled to elevated efflux of NH_4_^+^ from the root-tip area, and altered glycosylation of root proteins, resulting in an increase of unfolded proteins (Qin et al., 2008; Tanaka et al., 2015; Li et al., 2022a). In both mechanisms, the Vitamin C1 (VTC1) and Low Phosphate Root 2 (LPR2) genes play an important role. The VTC1 gene-mediated GDP-mannose synthesis function, rather than the ASA synthesis pathway, is considered key to the modification of protein glycosylation (Barth et al., 2010; Kempinski et al., 2011). In support of this view, the GDP-mannose pyrophosphate hydrolase knock-out mutant GDP-D-mannose pyrophosphohydrolase (NUDX9), impaired in mannose hydrolysis in Arabidopsis roots, showed significantly higher GDP-mannose levels and increased N-glycosylation of proteins (Di et al., 2021). Downstream of VTC1 in the GDP-mannose pathway is dolichol phosphate mannose synthase (DPMS1), which catalyses the biosynthesis of dolichol phosphate mannose (Dol-P-Man, the polyprenyl monosaccharide carrier, is the source of all mannose residues on glycoproteins within the endoplasmic reticulum lumen.), required for N-glycosylation and/or glycosylphosphatidylinositol (GPI)-anchored proteins (GPI-anchored proteins regulate cellulose deposition, wall integrity, membrane rafts, root growth and other development aspects.) (Jadid et al., 2011). Increased energy consumption associated with elevated NH_4_^+^ efflux in the root-tip zone is an important factor contributing to NH_4_^+^ toxicity. Root-tip-zone NH_4_^+^ efflux in the VTC1 mutant is indeed significantly increased. However, the mechanism by which VTC1 regulates NH_4_^+^ efflux in the root-tip zone remains unclear, and this is related to the fact that, hitherto, the channel(s) or carrier(s) responsible for transmembrane efflux of NH_4_^+^ have not been definitively identified. In rice, the transcription factor OsEIL1 directly induces the expression of *OsVTC1-3* to reduce NH_4_^+^ efflux under high-NH_4_^+^ conditions (Li et al., 2022a). Recently, two studies (Liu et al., 2022a, b) found that NH_4_^+^-regulated LPR2-mediated aberrant Fe accumulation impairs Arabidopsis root growth. The reports suggest that Fe plays a role in the regulation of root elongation and NH_4_^+^ efflux under NH_4_^+^ nutrition (Liu et al., 2023a; Li et al., 2024b).

Furthermore, reactive oxygen species (ROS) exert a strong influence as signaling molecules by adjusting the balance of PR cell proliferation and cell differentiation in root meristems. Total ROS levels were not found to be different between whole wild-type plants and *vtc1-1* mutant seedlings. However, the root apical meristem (RAM) of *vtc1-1* clearly exhibited higher H_2_O_2_ levels in the initiation and proximal zones when compared with wild type (Kka et al., 2018). An increase of the H_2_O_2_ level in the root meristem decreases meristem size and root elongation. Therefore, further exploration is needed to elucidate the local regulation of ROS levels by VTC1 in the root-tip zone and the participation in the response of PR growth to NH_4_^+^ toxicity. In rice, the heme oxygenase OsSE5 modulates NH_4_^+^ toxicity responses. OsSE5 mutant reduces APX, CAT, SOD activities, while its overexpression enhances ROS-scavenging enzyme activities, improving NH_4_^+^ stress tolerance in rice and Arabidopsis (Xie et al., 2015). Furthermore, kinase OsSAPK9 and transcription factor OsbZIP20 function as downstream components in ABA-mediated NH_4_^+^ detoxification, exerting their effects by reducing ROS and free NH_4_^+^ (Sun et al., 2020). The phosphorylation of plasma membrane proteins also plays an important role in the NH_4_^+^ tolerance of roots. The Ammonium transporter 1 (AMT1) protein is an important component of the NH_4_^+^ absorption system (Glass et al., 2002; Ma et al., 2025). When the concentration of NH_4_^+^ increases, AMT1 needs to close in time, to avoid excess NH_4_^+^ being absorbed by the root tissues. The expression of calcineurin B-like (CBL) interacting protein kinase 23 (CIPK23) is induced by an increase in external NH_4_^+^ concentration, and it has been shown to directly bind to AMT1.1 and AMT1.2 proteins and phosphorylate them, leading to the closure of the AMT1.1 and AMT1.2 transporters, thus reducing the entry of NH_4_^+^ (Straub et al., 2017). In rice, OsCIPK18 affects the NH_4_^+^ uptake by regulating the expression of *OsAMT1;2* (Sun et al., 2022). Hao et al. (2020) suggested that three root-expressed AMTs (*ZmAMT1.1a*, *1.1b*, or *ZmAMT1.3*) of maize engaged NH_4_^+^ uniporting as NH_4_^+^ uptake mechanisms. In wheat, *TaAMT1;1a*, *TaAMT1;1b*, *TaAMT1;3a*, and *TaAMT3;3a* exhibit higher expression levels in roots (Li et al., 2017). A mutation of CIPK23 resulted in increased absorption of NH_4_^+^, a higher cellular NH_4_^+^/K^+^ ratio, and increased sensitivity of PR growth to NH_4_^+^ (Coleto et al., 2023).

Auxin plays a critical role in root development, as both excessive and insufficient auxin levels in the root-tip zone impair PR elongation. However, current findings on the role of auxin in PR growth under ammonium stress are controversial. For example, a mutation of the auxin influx carrier Auxin resistant 1 (*AUX1*), which reduces root auxin levels, was reported to enhance PR tolerance to NH_4_^+^ (Cao et al., 1993). Consistent with this, Gao et al. (2020) demonstrated that exogenous auxin increases PR sensitivity to high NH_4_^+^, with *WRKY23* acting as a key transcription factor in the stress response. Under high NH_4_^+^, the root tip of the *wrky23* mutant accumulated more auxin than wild-type plants, leading to heightened PR sensitivity. By contrast, Yang et al. (2015) showed that auxin-regulated gene expression is globally repressed in Arabidopsis under high-NH_4_^+^ stress, and impaired PR growth on (NH_4_)_2_SO_4_ supplemented with NO_3_^-^ was partially rescued by exogenous auxin and in specific auxin-pathway mutants. A potential explanation for these divergent observations lies in the presence or absence of nitrate nitrogen in the high-NH_4_^+^ treatment medium. Nitrate is well known to alter the degree to which ammonium toxicity manifests (Kronzucker et al., 1999; Britto and Kronzucker, 2013). Qin et al. (2011) found that auxin levels in the Arabidopsis root tip (indicated by direct repeat 5 (*DR5*) and indole-3-acetic acid (*IAA5*) expression) increased over time under NH_4_^+^ plus NO_3_^-^ treatment but decreased under sole NH_4_^+^ treatment.

There are significant differences in the degree of NH_4_^+^ toxicity on root growth among different species. Under the treatment of 1.5 mM NH_4_^+^, the root biomass of spinach decreases by approximately 70%, while that of pea is not significantly affected (Dominguez-Valdivia et al., 2008). There are also obvious differences in the degree to which root growth is affected by NH_4_^+^ toxicity, which is controlled by different genes. For example, the mutation of the *GSNOR* gene in Arabidopsis leads to a roughly 30% increase in the sensitivity of roots to NH_4_^+^ toxicity compared with the wild type (Zhang et al., 2021a), whereas under the same degree of NH_4_^+^ toxicity, the mutation of the *WRRY23* gene in Arabidopsis only causes an increase of about 15% (Gao et al., 2020). In addition, the functions of homologous genes vary among different species. For instance, the mutation of the *EIN3* gene in Arabidopsis results in obvious resistance to NH_4_^+^ toxicity, with the tolerance increasing by approximately 20% compared with the wild type (Li et al., 2022a), while the mutation of the *OsEIL1* gene in rice enhances NH_4_^+^ sensitivity, with the sensitivity being about 15% higher than that of the wild type rice (Li et al., 2022a).

### Lateral root formation

LR elongation is suppressed by ammonium in a manner similar to the suppression of PR growth, yet lateral root formation can also be enhanced under local ammonium supply. Indeed, both LR initiation and higher-order LR branching are promoted by localized NH_4_^+^. NH_4_^+^-dependent LR branching is nearly absent in the Arabidopsis quadruple AMT knockout line *qko* (*amt1;1 amt1;2 amt1;3 amt2;1*), and only *AMT1;3* rescues ammonium-induced LR branching in *qko*. These findings suggest that NH_4_^+^ acts as a signaling molecule to activate LR formation, with sensor function potentially mediated by *AMT1;3* (Li et al., 2014; Liu and von Wiren, 2017). However, a recent study by Meier et al. (2020) demonstrated that local ammonium supply stimulates auxin accumulation in the root vascular system and promotes LR formation, leading to a highly branched root system. Mechanistically, measurements of pH dynamics and of auxin activity indicate that the acidification of the root apoplastic space associated with ammonium-transporter-mediated NH_4_^+^ uptake (Glass et al., 2002; Britto and Kronzucker, 2005) enhances the uptake of protonated auxin into cortical and epidermal cells overlying LR primordia, thereby promoting LR formation. This process allows auxin to reach LR primordia independently of the auxin influx carriers *AUX1* and Like-AUX1 (*LAX3*), via NH_4_^+^-induced and H^+^-ATPase-mediated apoplastic acidification (Meier et al., 2020). The authors propose that, rather than NH_4_^+^-mediated signal transduction, NH_4_^+^-uptake-dependent pH reduction in the apoplast facilitates enhanced radial auxin diffusion to LR primordia, and this drives LR branching. In rice, upon newly developed roots accessing NH_4_^+^-rich soil patches, NH_4_^+^ induces the expression of *OsAMT1;1* and *OsAMT1;2*, which transiently enhances the capacity for high-affinity NH_4_^+^ uptake and triggers LR development to further explore the soil region (Wu et al., 2022).

Notably, LR emergence is regulated by shoot-derived, not root-derived, ammonium signals (Li et al., 2011). Long-term root exposure to high NH_4_^+^ concentrations leads to NH_4_^+^ accumulation in shoots, which enhances shoot ethylene production (Li et al., 2013). This process reduces LR formation by suppressing *AUX1* expression in shoots. Shoot-localized *AUX1* downregulation decreases auxin influx and subsequent auxin levels, thereby impairing *AUX1*-dependent long-distance auxin transport and auxin responses in roots (Li et al., 2013, 2014). Collectively, NH_4_^+^ effects on LR formation involve systemic signaling, while local signals govern LR initiation. In rice, both *OsAUX1* and *OsAUX3* are involved in the regulation of PR elongation and LR development (Zhao et al., 2015; Wang et al., 2019). Under short-term localized high-NH_4_^+^ conditions, NH_4_^+^ uptake acidifies the apoplast (via H^+^-ATPase activity), enhancing protonated auxin diffusion to LR primordia and promoting LR initiation (Meier et al., 2020). This short-term response benefits root systems by facilitating local nitrogen acquisition to withstand stresses such as waterlogging. Conversely, under long-term ammonium exposure, shoot NH_4_^+^ accumulation triggers ethylene production, which inhibits *AUX1* function. This reduces shoot-to-root auxin transport and LR-primordium auxin responses, ultimately suppressing LR formation. Vacuolar ammonium compartmentation has been identified as an ammonium-sensitive target for the receptor-like kinase Ca^2+^-associated protein kinase 1 (*CAP1*) (Engelsberger and Schulze, 2012; Bai et al., 2014). In the Arabidopsis *cap1-1* mutant, root hair elongation is highly sensitive to high NH_4_^+^, linked to disrupted cytosolic Ca²^+^ gradients in root hairs.

## Responses of roots to Fe toxicity

The critical redox potential for Fe reduction and subsequent dissolution are –100 mV at pH 8, and between +300 mV and +100 mV at pH 6 and 7, while appreciable reduction occurs at +300 mV at pH 5 (Parent et al., 2008; Becker and Asch, 2005). Iron toxicity is a nutritional disorder syndrome that is typically associated with large concentrations of reduced iron (Fe^2+^) in the soil solution. Fe^2+^ is increasingly present in lower soil strata, where low pH and hypoxia or anoxia prevail in waterlogged soil (Parent et al., 2008). The extent of the oxidized surface layer can vary from 0.2 to 1 cm, partly determined by nitrate-dependent microbial reoxidation of Fe^2+^ (Zahra et al., 2021). The highest Fe^2+^ concentrations are found at soil depths of 2–15 cm, which covers the majority of the root system of most crops (Aung and Masuda, 2020; Li et al., 2024a). Oxidation-reduction affects the valence of Fe and thereby its uptake by plants. Oxygenation of the rhizosphere to exclude Fe represents a primary adaptive mechanism in Fe-toxic soils (Becker and Asch, 2005). Variations in Fe^2+^ exclusion capacity exist among plant genotypes, attributed to differences in aerenchyma development, barriers to radial O_2_ loss, and enzymatic Fe^2+^ oxidation activity. Fe^2+^ that penetrates the rhizospheric oxidation zone enters the root apoplast via diffusion and mass flow within the transpiration stream (Li et al., 2024a). For transport to the shoot via the xylem, apoplastic Fe^2+^ must traverse cell membranes into the symplasm to bypass the Casparian strip in the endodermis, which constitutes a potential exclusion mechanism, at least in undamaged roots (Kirk et al., 2022). In healthy roots, the majority of absorbed Fe^2+^ is retained in metabolically inactive forms, likely involving sequestration in root cell vacuoles (Moore et al., 2014) and in plastid-localized ferritin proteins. Furthermore, excess Fe can arrest PR growth in Arabidopsis and inhibit LR initiation in newly grown roots elongating during the exposure to excess Fe, sparing proximal roots formed prior to the imposition of excess Fe, resulting from direct contact of the root tip with excess Fe (Li et al., 2016, 2024a). As soon as the root tips experiences more intense iron exposure, the PR and LR in newly grown roots weaken or stop growing. This adjustment of RSA is a sensible response to limit excess absorption of Fe from the lower soil strata under waterlogging, and, in this manner, more severe iron toxicity can be prevented while still allowing the remainder of the root system to obtain essential nutrients and O_2_ in the upper soil strata. This strategy is further helped by that fact that excess iron is thought to have no significant effect on proximal roots, LR formation is relatively stable in terms of both the number and length of LRs in this part of the root system, while the absorption of other nutrients and O_2_ can be delegated to other components of the root system, allowing intelligent acclimation to nutrient and O_2_ pressures.

### Primary root growth

Given that root elongation rates are regulated by cell division and expansion along the root’s longitudinal axis, a critical question is how Fe toxicity impacts this growth and which cellular process serves as the primary target. Experiments delivering excess Fe to distinct root zones in Arabidopsis have shown that direct contact between the root tip and elevated Fe is both necessary and sufficient to inhibit PR elongation (Li et al., 2015). Excess Fe arrested PR-tip growth by reducing both cell elongation and division. Notably, Fe toxicity did not affect the G_2_-to-M transition in the cell cycle of the root-tip zone. Ethylene, a well-established regulator of root development, has long been linked to Fe-mediated inhibition of root elongation. Evidence includes the observation that ethylene-overproducing mutants (e.g. *eto1*) or signal-enhanced mutants (e.g. constitutive triple response 1 (*ctr1*)) exhibit less Fe-induced root length reduction compared to wild-type plants, whereas the ethylene-insensitive *etr1* mutant shows heightened sensitivity to excess Fe (Li et al., 2015). By contrast, abscisic acid (ABA) and auxin—two key stress-response hormones—do not appear to be involved in Fe-mediated inhibition of PR growth (Li et al., 2016).

Excess Fe modulates the H_2_O_2_/O_2_^-^ balance, decreasing O_2_^-^ in the root-tip proliferation zone while increasing H_2_O_2_ production in the transition zone, thereby arresting PR growth (Reyt et al., 2015). This aligns with the model proposed by Tsukagoshi et al. (2010), which proposes a correlation between PR growth with the relative distribution of O_2_^-^ and H_2_O_2_ mediated by UPB1 (UPBEAT1, a transcription factor) in the root tip. While ethylene and ROS signaling are known to interact in various abiotic stresses, whether ethylene acts independently or in conjunction with ROS during root-tip acclimation to Fe excess remains unresolved.

Additionally, enhanced nitric oxide (NO) generation, for example in the NO-overproducing *nox1* (nitric oxide overproducer 1) mutant, has been shown to promote root growth inhibition under Fe toxicity (Zhang et al., 2018). NO levels are significantly elevated in root tips compared to other root regions, and Fe-induced growth arrest is at least partially linked to NO-mediated K^+^ efflux, potentially via the activity of SNO1 (1 (sensitive to nitric oxide 1))/SOS4 (salt overly sensitive 4)-dependent non-selective cation channels (NSCC, these channels exhibit weak cation selectivity; additionally, some of them are permeable to divalent cations and may even allow anion conduction under certain conditions.). Ethylene partially counteracts Fe-mediated PR growth inhibition by regulating NO levels.

Root elongation is widely used as a phenotypic indicator of plant adaptation to environmental stress, and its genetic regulation under stress conditions has garnered significant research interest. Given the substantial variation in Fe sensitivity among crop and wild plant species, identifying genetic loci controlling this trait has been a focus of recent efforts. Using genome-wide association studies (GWAS), GSNOR (*S*-nitrosoglutathione (GSNO) reductase1), an enzyme critical for metabolizing GSNO and maintaining NO homeostasis, was recently identified as a major gene underlying Arabidopsis tolerance to Fe-mediated toxicity (Li et al., 2019). Notably, GSNOR is essential for root tolerance to Fe toxicity not only in Arabidopsis but also in other dicots like Lotus japonicus and the monocot rice. Studies have shown that GSNOR-regulated NO accumulation is a key contributor to Fe- and H_2_O_2_-induced oxidative damage in plants (Li et al., 2021). Similar to Arabidopsis, OsGSNOR knockout lines displayed slightly reduced root growth under control conditions, but were dramatically more sensitive to Fe toxicity compared to the rice wild-type (Li et al., 2021). Further research reveals that GSNOR responds to the problem of iron toxicity in plant roots by regulating a molecular signaling pathway for root-stem cell death composed of mitochondria-VDAC-DNA damage-ANAC044 (Yan et al., 2025). However, the NO-overproducing *nox1* mutant exhibited sensitivity to Fe toxicity with excessive NO accumulation, yet its ROSlevels did not differ from the wild type. This suggests that GSNOR may play a NO-independent role in mitigating iron-induced oxidative stress.

There are significant differences in the extent to which root growth of different species is affected by Fe toxicity. Under the treatment with 350 μM Fe concentration, the root elongation of wild type Arabidopsis decreases by approximately 15%, that of wild type rice decreases by about 8%, while the root biomass of *L. japonicus* is not significantly affected (Li et al., 2019). There are also obvious differences in the degree to which root growth responses to Fe toxicity are regulated by different genes. For example, the mutation of the *AUX1* gene in Arabidopsis increases the sensitivity of PR elongation to Fe toxicity by approximately 20% compared with the wild type, and the mutation of the *PIN2* gene increases such sensitivity by about 11% (Li et al., 2015). Under the same level of Fe toxicity, the mutation of the *AUX1* gene in Arabidopsis enhances the sensitivity of LR number by around 45%, while the mutation of the *PIN2* gene increases such sensitivity by about 20% (Li et al., 2015). In addition, there are differences in the functional extent of homologous genes among different species. For instance, the mutation of the *GSNOR* gene in Arabidopsis leads to significant sensitivity to Fe toxicity during growth, with the sensitivity increasing by approximately 40% compared with the wild type (Li et al., 2019). The mutation of the *OsGSNOR* gene in rice increases the sensitivity by about 20% compared with wild type rice, and the mutation of the *LjGSNOR* gene in *L. japonicus* enhances the sensitivity by around 30% compared with its wild type (Li et al., 2019).

### Lateral root formation

The root tip also serves as the primary sensing site for LR formation in response to excess Fe. Reduced LR formation under excess Fe has been partially linked to auxin levels, while root-tip PIN2 protein expression and ethylene-related AUX1 function play positive roles in LR formation under Fe excess (Li et al., 2015). Additionally, LR development requires ROS signaling (Manzano et al., 2014). Excess Fe increases ferritin abundance, facilitating Fe storage and protecting plants from Fe-induced oxidative stress. In *fer1-3-4* (ferritin) plants, the Fe-excess-mediated reduction in LR length and density is exacerbated due to a defect in LR emergence. Arabidopsis seedlings exposed to oxidative stress-inducing agents exhibit altered auxin homeostasis, suggesting crosstalk between ROS and auxin signaling (Cheng et al., 2011; Yuan et al., 2013). This may represent a signaling hub in the root tip to mediate adaptive growth responses to excess Fe.

The PIN2 gene, critical for polar auxin transport to both root and shoot apices, is a general stress target due to its sensitivity to diverse environmental stresses (e.g. cold, salt, and aluminum), supporting root stress avoidance (Baluska et al., 2010). Under Fe stress, auxin distribution in the root responds to changes in PIN2 gene expression in the root tip (Li et al., 2015), potentially redirecting root growth away from the stress stimulus (Sun et al., 2008). Furthermore, ROS and NO signaling in the root apex are involved in early gravitropic acclimation responses (Mugnai et al., 2014). Hypoxic or anoxic conditions increase Fe²^+^ availability (Mongon et al., 2014), and the transition zone is the most oxygen-deprivation-sensitive region of the root (McLamore et al., 2010; Larsen et al., 2015). Local NO peaks in the transition zone are essential for the root’s acclimation to oxygen deprivation (Mugnai et al., 2012).

**Figure 2:**
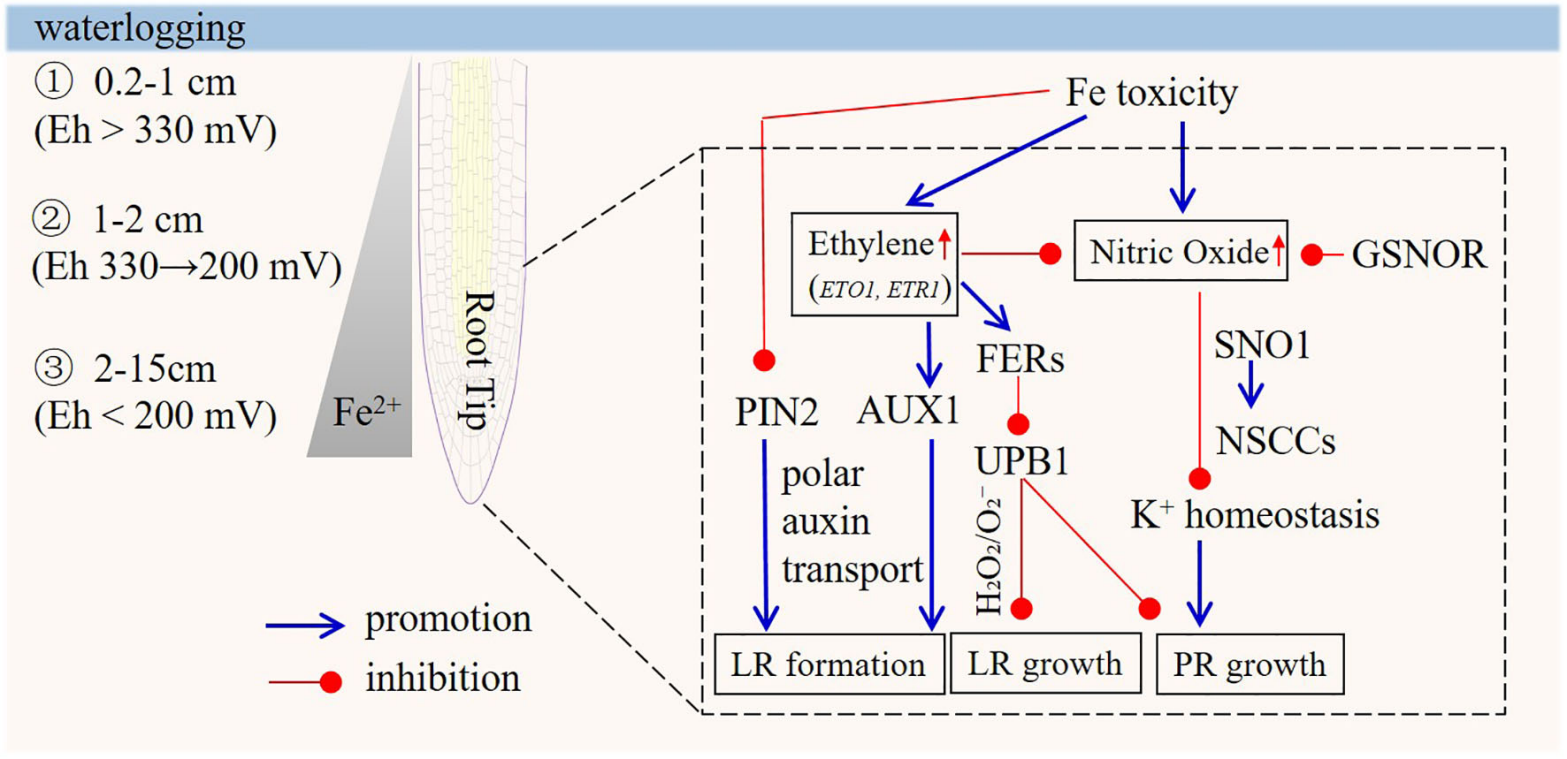
Model of the signaling responses under iron (Fe) toxicity in the root tips. Iron toxicity, caused by high soil-solution Fe²^+^, is common in waterlogged soil. The oxidized surface layer ranges from 0.2 to 1 cm, and Fe²^+^ peaks at 2–15 cm, affecting plant roots as redox reactions impact Fe valence and uptake. In Arabidopsis, direct contact between root tips and excess Fe inhibits primary root elongation by reducing cell division and elongation, without affecting the G_2_-M transition. Ethylene is involved, as ethylene-related mutants show varying sensitivities to Fe toxicity. Excess Fe also modulates H_2_O_2_/O_2_^-^ balance and NO levels, with ethylene countering growth inhibition by regulating NO. The GSNOR gene is key for Fe tolerance across species, regulating a pathway related to root-stem cell death. Lateral root formation is impacted indirectly, via the root tip, which senses excess Fe. Reduced formation links to auxin, while the PIN2 and AUX1 genes play positive roles. ROS signaling is essential, and excess Fe increases ferritin. The PIN2 gene affects auxin distribution under Fe stress, potentially redirecting root growth.

Despite progress in understanding molecular determinants of root sensitivity to Fe toxicity (**Figure 2**), key questions remain: What pathway mediates Fe toxicity-induced NO production in the apical region, given that current evidence does not support a NO-synthase-dependent pathway? How do ethylene and ROS interact to regulate PR elongation under Fe toxicity? Additionally, what are the primary signaling molecules or genes in the apical region responsible for the sensing of Fe stress?

## Responses of roots to Mn toxicity

Oxidation–reduction status is the best single indicator of the degree of anaerobiosis in the flooded soil, and allows reasonable predictions to be made concerning the behavior of several essential plant nutrients. Oxygen is used within the first hours or days after waterlogging, and the subsequent reduction of Mn^4+^ also occurs rapidly, since the content of manganese in the soil is usually low. Manganese (II) is the most soluble form of manganese, so high concentrations of Mn^2+^ will occur in soil solutions under waterlogged conditions. High concentrations of Mn^2+^ in waterlogged soils have been named as a major constraint for growing sensitive wheat cultivars in areas prone to waterlogging in Australia and India. If flooding occurs under acidic soil conditions, soil manganese activity will increase more significantly (Khabaz-Saberi et al., 2010b; Huang et al., 2015). When the pH is ~5, almost all soil manganese changes from the reducible state to the water-soluble and exchangeable state, even when the Eh is as high as + 500 mv. At a pH of 6-8, the conversion takes place at relatively lower redox potentials of +200 to +300 mV. Excess Mn significantly reduces the growth of the Arabidopsis PR, and the decrease of PR elongation has been shown to be positively correlated with Mn concentration. Excess Mn inhibits PR growth and altered patterns of auxin accumulation and PR distribution are documented in Mn-treated roots (Zhao et al., 2017).

**Figure 3:**
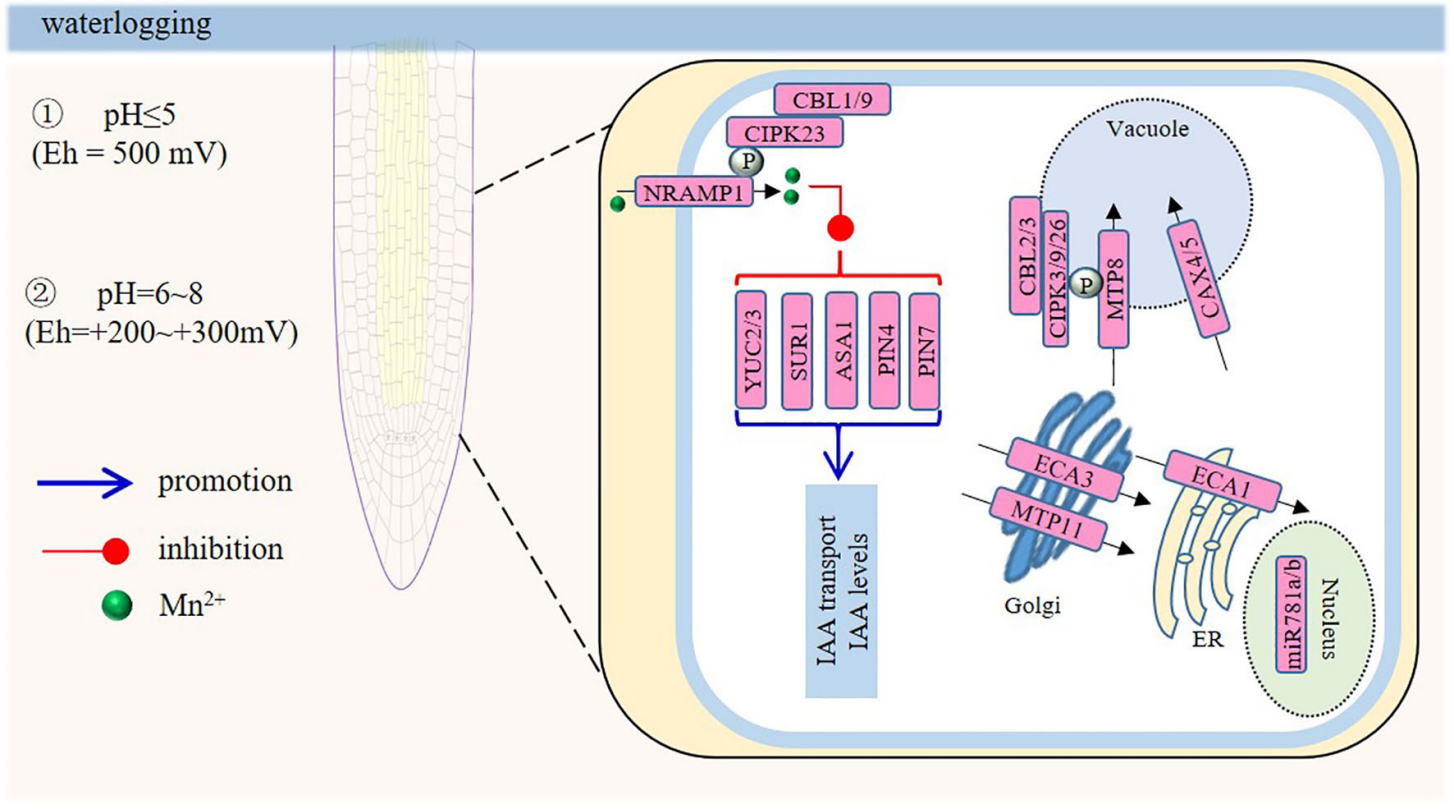
Model of the signaling responses in roots under manganese (Mn) toxicity. When the pH is at 5, almost all soil manganese changes from the reducible state to the water-soluble and exchangeable states, even when the Eh is as high as + 500 mV. At a pH of 6-8, the conversion takes place at relatively lower redox potentials of +200 to +300 mV. Mn toxicity inhibits primary root (PR) elongation by reducing meristem-cell mitosis. Auxin is central: Mn toxicity decreases root-tip auxin levels by repressing auxin-biosynthesis genes such as YUC2, YUC3, SUR1, and ASA1, and by down-regulating the efflux carriers PIN4 and PIN7; pin4/pin7 mutants show less root-growth inhibition. MicroRNAs also play roles; miR781a and miR781b, involved in root development, are upregulated by excess Mn. Among transporters, CAX4, expressed in the root apex and in lateral root primordia, is crucial; *cax4-1* and *CAX4 RNAi* seedlings have reduced root growth under high Mn²^+^. MTP11 detoxifies Mn; the *mtp11-1* mutant is Mn-sensitive, and overexpression confers tolerance. MTP8 is a tonoplast Mn²^+^ transporter vital for primary-root Mn tolerance. AtECA1 and AtECA3 affect root-hair elongation under high Mn. Additionally, the CBL1/9–CIPK23 and CBL2/3–CIPK3/9/26 complex phosphorylates NRAMP1 or MTP8, respectively, enhancing high-Mn tolerance.

Most studies on the physiological mechanism of the toxicity response to Mn have focused on Mn-mediated inhibition of photosynthesis, Mn-mediated changes in antioxidant enzymes, and the production of ROS (Shao et al., 2017). However, studies on the root physiology in response to Mn toxicity have been rare. Mn toxicity inhibits PR elongation by decreasing the mitotic potential of meristem cells (Zhao et al., 2017). Auxin plays a central role in regulating PR growth and development under Mn toxicity. Mn toxicity can reduce auxin levels in root tips by reducing IAA biosynthesis, and the expression of auxin biosynthesis-related genes, such as YUC2 (YUCCA2), YUC3, SUR1 (Superroot1), and ASA1 (Anthranilate synthase alpha subunit 1), have been shown to be significantly repressed by Mn toxicity (Zhao et al., 2017). Mn toxicity also down-regulates the expression of the genes encoding the auxin-efflux carriers PIN-FORMED 4 (PIN4) and PIN7 (Zhao et al., 2017). Loss of function *pin4* and *pin7* mutants show less inhibition of root growth than Arabidopsis *Col-0* seedlings. MicroRNAs (miRNAs) play key roles in regulating Mn-toxicity tolerance in plants, and Mn toxicity may also regulate root developmental processes by regulating miRNA expression. Several miRNAs involved in modulating root system development (e.g. miR781a and miR781b) have been implicated in the Mn-toxicity response in Arabidopsis roots, by using small-RNA sequencing (Gong et al., 2019). miR781 modulates root meristem function and is essential to embryo development (Sabelli et al., 2009), and two miR781 genes, miR781a and miR781b, were upregulated in excess-Mn-treated Arabidopsis seedlings. Cation exchangers (CAXs) play a key role in mediating cation influx into the vacuole. CAX4 is expressed in the root apex and LR primordia, and that expression is increased when Mn^2+^ levels are elevated. Under high-Mn^2+^ conditions, both PR length and LR number of *cax4-1* and *CAX4 RNAi* seedlings were reduced significantly compared to control seedlings (Mei et al., 2009). While direct mechanistic studies on Mn²^+^ toxicity-induced changes in LR development still be limited, *CAX4* expression in LR primordia may provide emerging links to LR modulation under Mn toxicity. Furthermore, ASA1 and YUC2, which can be repressed by Mn toxicity, are involoved in regulating the occurrence of LR (Zhang et al., 2023a). All these provide valuable clues for future research on the regulation of LR development by Mn toxicity. In addition, there was an increase in CAX5 transcripts under conditions of excess Mn (Edmond et al., 2009), but the role of CAX5 in the Mn-toxicity response, especially in roots, is still unclear. The Arabidopsis CDF protein Metal tolerance protein 11 (MTP11) plays an important role in the detoxification of Mn. *ProAtMTP11:GUS* showed a high activity in root tips, and the *Arabidopsis mtp11-1* mutant is hypersensitive to excess Mn, whereas an MTP11 overexpressor is excess-Mn-hypertolerant (Delhaize et al., 2007; Peiter et al., 2007). The increased sensitivity of *mtp11-1* may be related to excessive accumulation of Mn^2+^ (Peiter et al., 2007). Similar roles of other MTPs in sequestering Mn into the Golgi apparatus have been documented for OsMTP11 from rice (Tsunemitsu et al., 2018). The MTP8, a member of the Cation Diffusion Facilitator family, was characterized as a Mn^2+^ transporter localized in the tonoplast (Eroglu et al., 2016, 2017; Chu et al., 2017). AtMTP8 expression was increased on excess-Mn^2+^ media and was a critical determinant for excess-Mn tolerance of PR growth (Eroglu et al., 2016). Ju et al. (2022) suggested CBL2/3 and CIPK3/9/26 negatively regulates vacuolar Mn transporter MTP8 transport activity via phosphorylation and its consequences for high-Mn tolerance in Arabidopsis. In Arabidopsis, the ECA (ER-type Calcium ATPase) subfamily has two members, AtECA1 and AtECA3, involved in endomembrane-Mn^2+^ transport (Huda et al., 2013). On high-Mn^2+^ media, root-hair elongation was inhibited in the *eca1* mutant, suggesting an impairment in tip growth (Wu et al., 2002), possibly by linkage to the regulation of Ca^2+^ pumps. Zhang et al. (2023b) identified a CBL (calcineurin B-like) 1/9–CIPK23 complex that is critical for the phosphorylation of NRAMP1 (Natural resistance-associated macrophage protein 1). The Ca^2+^ signal induced by Mn toxicity activates the CBL1/9–CIPK23 module, which phosphorylates Ser20 of NRAMP1, promoting the endocytosis of NRAMP1 under high-Mn stress, and then increasing high-Mn tolerance of plants (**Figure 3**). In rice, Mn uptake is mediated by OsNRAMP5, a homolog of NRAMP1 in Arabidopsis (Sasaki et al., 2012). Similarly, ZmNRAMP2 in maize facilitates the translocation of Mn from roots to seedlings by accelerating the release of vacuolar Mn in xylem parenchyma cells (Guo et al., 2022). Wang et al. (2023) revealed the key role of TaNRAMP3 in Mn transport in Triticum aestivum, allowing it to adapt to Mn-deficiency stress. Furthermore, RNA sequencing (RNA-seq) was employed to examine differentially expressed genes (DEGs) in soybean roots under Mn toxicity. Among these DEGs, 572 were upregulated and 858 were downregulated, suggesting that soybean roots may activate complex molecular regulatory mechanisms in response to Mn toxicity (Liu et al., 2023b).

## Conclusion and future prospects

**Figure 4:**
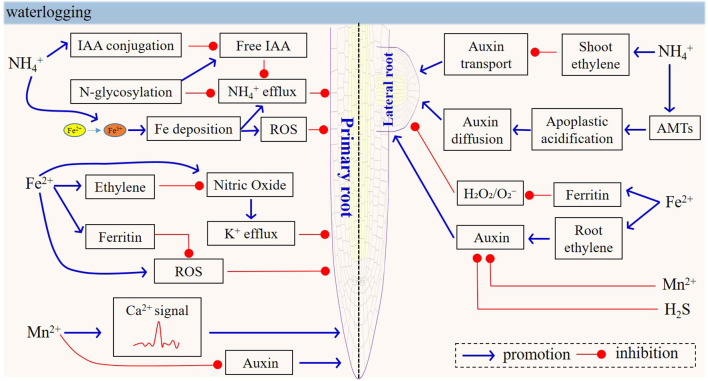
Model of physiological and signaling responses in roots under NH_4_^+^, Fe^2+^, Mn^2+^ and H_2_S toxicity. In natural waterlogged soils, multiple ion toxicities—including NH_4_^+^, Fe²^+^, Mn²^+^, and H_2_S—often co-occur, and hormones (e.g., auxin and ethylene), NO, AMTs, ROS, and Ca^2+^ have been identified to play crucial roles in mediating Fe-, Mn-, and NH_4_^+^-toxicity signals as well as in regulating the balance between root growth and stress responses, thus summarizing the physiological and signaling responses induced by various toxicities.

Since the 1990s, a number of studies have shown the relationship between ion toxicity (e.g. Fe and Mn) and waterlogging stress (Stieger and Feller, 1994; Ding and Musgrave, 1995; Setter et al., 2004), raising the hypothesis that ion toxicity in soil may be the key factor determining waterlogging tolerance (Setter et al., 2004, 8^th^ Conference of the International Society for Plant Anaerobiosis; Jackson and Colmer, 2005). After decades of research since that time, the role of ion toxicity in waterlogging and flooding tolerance has become more clear. Roots are the first targets for waterlogging-induced ion toxicity. The biological processes and mechanisms of ion (Fe, Mn, NH_4_^+^) toxicity have been reviewed, and the defense response of roots and the role of related genes have also been described. Fe, Mn, and NH_4_^+^ toxicity are widely described to reduce root growth and to affect plant development. The impact of Fe, Mn, NH_4_^+^ toxicity on the growth of individual root organs differs in severity, resulting in altered root morphology. Hormones (e.g. auxin and ethylene), NO, AMTs, transcription factors, and phosphokinases have been found to play important roles in mediating Fe-, Mn-, and NH_4_^+^-toxicity signals and in controlling the balance between root growth and stress responses (**Figure 4**). High levels of H_2_S also inhibit auxin transport by altering the polar subcellular distribution of PIN proteins, thereby leading to alterations in root system development (Jia et al., 2015). Sophisticated crosstalk occurs among the different signals in this process. Furthermore, we should mention that there are many inconsistent results, such as pertaining to the role of auxin in NH_4_^+^ tolerance of PR growth. In general, the analysis of signal-component mutants should permit more definitive conclusions, but it should also be pointed out that effects of such signals in the contexts of Fe-, Mn-, and NH_4_^+^-toxicity will depend on culture conditions. The identification of phenotypic traits with lower genetic complexity that are nevertheless highly correlated with elevated ion-toxicity tolerance will be key to the successful discovery of novel candidate genes and their alleles.

Understanding the adaptive mechanisms of plants to waterlogging-induced ion toxicity is essential for formulating strategies and technical measures to mitigate damage. First, the adoption of waterlogging-tolerant cultivars. Although there are few reports on the selection of plant varieties tolerant to waterlogging-induced ion toxicity, genotypic differences in waterlogging tolerance have been observed in several crops. For example, different rice varieties exhibit varying levels of resistance to Fe toxicity (Kirk et al., 2022). Therefore, it is feasible to identify or develop crop varieties with better tolerance to waterlogging-induced ion toxicity. However, given the limited efficiency of traditional screening and breeding methods, modern molecular tools may be more useful. Thus, the identification of genes responsive to waterlogging-induced ion toxicity will facilitate the advancement of this work. Second, foliar and soil fertilization. Timely application of K after waterlogging may partially alleviate the damage caused by waterlogging-induced ion toxicity. K homeostasis helps to mitigate NH_4_^+^ toxicity and Fe toxicity (Zhang et al., 2018; Coleto et al., 2023). Ashraf et al. (2011) demonstrated that K supplementation enhanced plant growth in cotton plants subjected to waterlogging. Therefore, foliar or rhizosphere application of K fertilizer is likely to be an effective measure to reduce losses in field crops after waterlogging.

Ion toxicities trigger early and short-term responses, involving perception and transduction of the stress signal, and subsequent long-term responses that involve remodelling of the transcriptional network to regulate growth. In most studies, the waterlogging treatment is relatively long (in excess of four weeks), whereas, in a smaller number of studies, very young plants were subjected to water-logging for only about two weeks. Although the temporal data on activation of individual signaling pathways is relatively well characterized, early signaling responses that occur within seconds to hours after ion exposure are often uncharacterized. The identification of Fe^2+^, Mn^2+^, and NH_4_^+^ sensors in plants remains one of the most important topics in this field. As both ion toxicities (such as the toxicities elicited by Fe^2+^, Mn^2+^, NH_4_^+^) and oxygen deprivation are critical under waterlogging, it is instructive that oxygen sensors have been found in both plants and animals. Therefore, complex communication and feedback mechanisms between and among ion and oxygen sensors can also be expected. Furthermore, when translating results into the field, it is crucial to consider not only the time at which the waterlogging occurs, but also the prevailing temperature. If waterlogging occurs during a warm spring or summer, it is likely that micronutrients will increase in the plant. Most of the current studies examining roots responses to ion toxicity have to date not considered temperature and were mostly focused on the study of a single ion toxicity. Advances in phenotyping software, permitting the complex analysis of root-growth dynamics, and the identification of novel morphological responses under multiple environmental conditions will result in a deeper understanding of the morphological reprogramming that takes place in response to waterlogging and its associated ion toxicities. Naturally, there are many other interesting questions worth investigating further, and we outline a few of these under ‘Outstanding Questions’ (see below).

### Outstanding questions

1. The root tip zone is the key site for sensing Fe and NH_4_^+^ toxicity in root systems. Is the key response site for Mn²^+^ also the root tip? Are there root-tip-zone-specific factors that perceive Fe- and NH_4_^+^-toxicity signals? Can the root tip zone serve as a key site to identify Fe and NH_4_^+^ sensors?
2. Current knowledge of root ion toxicity is predominantly focused on annual plants like Arabidopsis and rice, which have very short life spans. To what extent can findings from these annuals be extrapolated to species with alternative life histories and long-lived plant roots?
3. Research on the mechanisms of waterlogging-induced ion toxicity has largely focused on single ion toxicities. While this has been helpful for the observation of real-time changes in root signals, it is disconnected from actual waterlogging contexts. However, when studying ion toxicities in waterlogged soils, effective and timely observation and analysis of real-time root signal changes remains challenging. How to establish an effective experimental system remains one of the field’s core topics.
4. What is the threshold of ion concentrations that plant roots can tolerate under waterlogging conditions?
5. Ion-toxicity-mechanism research has focused primarily on single-ion toxicity analysis, with limited attention to the simultaneous effects of multiple ion toxicities. Additionally, as waterlogging duration and temperature conditions change, ion-toxic combinations can shift significantly. How do root signals respond to these dynamic changes?
